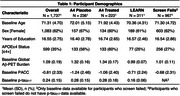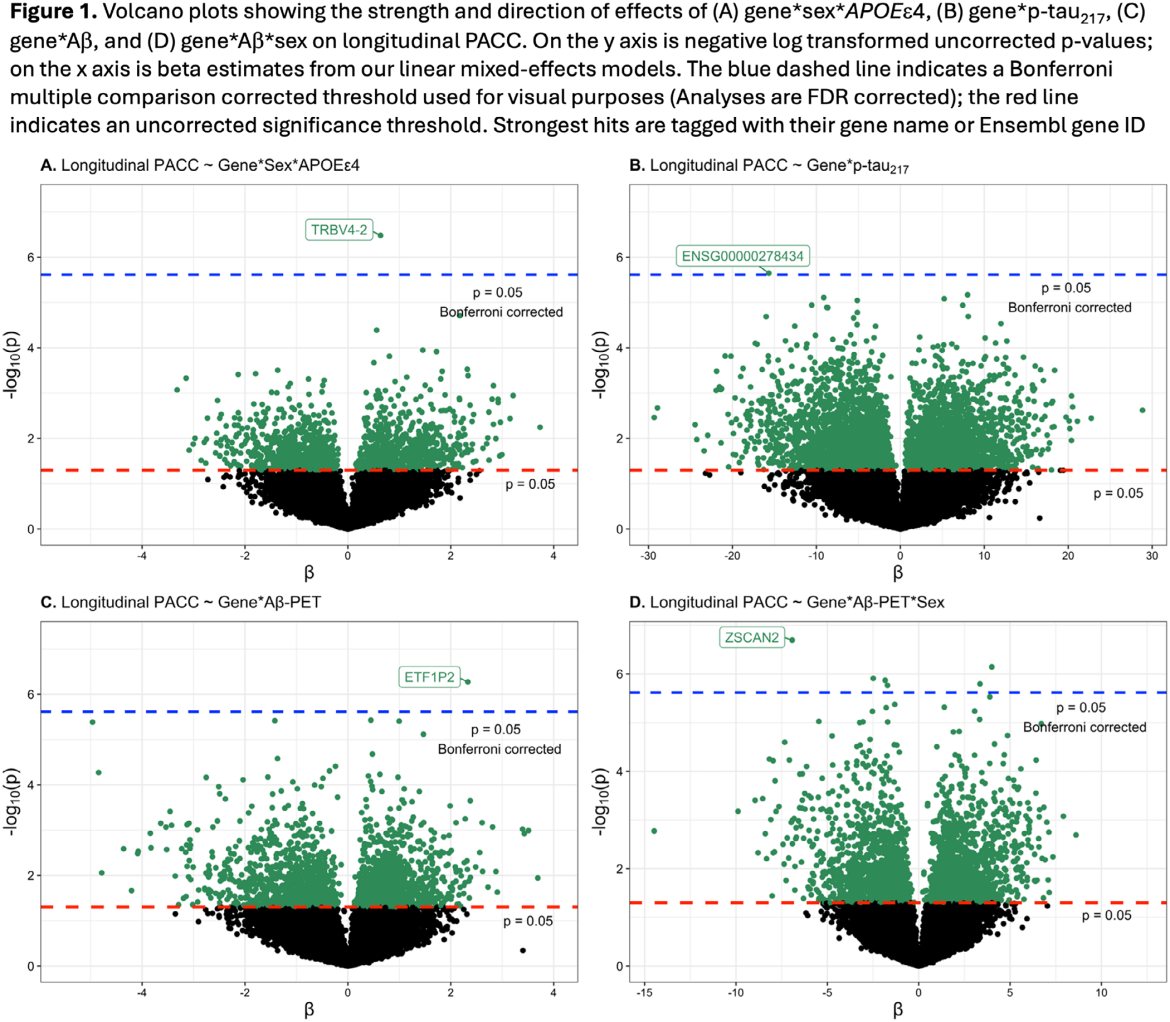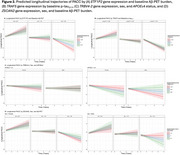# Whole blood gene expression associations with cognitive decline

**DOI:** 10.1002/alz70855_104370

**Published:** 2025-12-24

**Authors:** Hannah M Klinger, Vaibhav A Janve, Mabel Seto, Jane A Brown, Colin Birkenbihl, Gillian T Coughlan, Diana L Townsend, Michael J. Properzi, Jane Zyski, Ting‐Chen Wang, Rebecca E. Amariglio, Kathryn V Papp, Dorene M. Rentz, Hyun‐Sik Yang, Jasmeer P. Chhatwal, Michelle J. Clifton, Michael C. Donohue, Rema Raman, Robert A. Rissman, Paul S. Aisen, Keith A. Johnson, Reisa A. Sperling, Logan Dumitrescu, Timothy J. Hohman, Rachel F. Buckley

**Affiliations:** ^1^ Massachusetts General Hospital, Harvard Medical School, Boston, MA, USA; ^2^ Vanderbilt Memory and Alzheimer's Center, Vanderbilt University Medical Center, Nashville, TN, USA; ^3^ Brigham and Women's Hospital, Harvard Medical School, Boston, MA, USA; ^4^ Vanderbilt Memory & Alzheimer's Center, Vanderbilt University Medical Center, Nashville, TN, USA; ^5^ Alzheimer's Therapeutic Research Institute, University of Southern California, San Diego, CA, USA, San Diego, CA, USA; ^6^ Alzheimer's Therapeutic Research Institute, University of Southern California, San Diego, CA, USA; ^7^ Vanderbilt Memory and Alzheimer's Center, Vanderbilt University School of Medicine, Nashville, TN, USA

## Abstract

**Background:**

Bulk brain tissue studies of gene expression have discovered novel biological pathways associated with Alzheimer's disease (AD) neuropathology and pre‐morbid cognitive decline. These findings are primarily tuned to much older adults and are focused on end‐of‐life changes. The objective of this study was to ascertain transcriptomic signals from whole blood associated with cognitive decline in clinically unimpaired older adults from the A4 and LEARN studies. Identifying genes in whole blood that are associated with *in vivo* AD phenotypes can elucidate biological pathways implicated in the earliest disease processes.

**Method:**

1,737 participants were included from the A4 clinical trial of solanazumab. Both placebo and treatment arms were included (and participants who screen failed at baseline to inform our model intercepts), and the adjoining LEARN observational study (Aβ‐). All participants had whole blood gene expression data from the autosome and X chromosome (20,621 genes), and 770 participants (71.3±4.7 years; 62% Female; 45% *APOE*ε4+; 59% Aβ+) had at least two cognitive assessments measured using the PACC. Linear mixed‐effects models determined the association between gene expression alone and interactions with sex, *APOE*ε4, continuous Aβ‐PET burden, and *p*‐tau_217_ on longitudinal PACC. All analyses included random intercepts/slopes, and adjusted for age, education, cohort, PACC version, and cumulative dose. All analyses were FDR corrected.

**Result:**

No individual transcript was associated with longitudinal cognition after FDR correction. 167 genes were associated with longitudinal cognition when modified through different markers. One gene (*TRBV4‐2*; Figure 2C) was moderated by *APOE*ε4 x sex, 6 genes by Aβ‐PET, 118 genes by Aβ‐PET x sex, and 17 genes weakly by *p*‐tau_217_ (Figure 1). Of note, lower *ETF1P2* expression, a noncoding pseudogene that resembles *ETF1* and plays a role in protein synthesis, was associated with faster cognitive decline among participants with high baseline Aβ‐PET burden (Figure 2A; b_INT_=2.33(0.456), *p_FDR_* = 0.011). Similarly, greater *ZSCAN2* expression, implicated in regulating inflammation, was associated with faster cognitive decline particularly among males with higher baseline Aβ‐PET burden (Figure 2D; b_INT_=‐6.92(1.33), *p_FDR_* = 0.004).

**Conclusion:**

Whole blood transcriptomic signals were primarily associated with cognitive decline via interactions with Aβ‐PET, Aβ‐PET x sex, and *p*‐tau_217_. Further work, such as enrichment analysis and validation in external cohorts, will elucidate relevant biological pathways.